# CHL1 and NrCAM are Primarily Expressed in Low Grade Pediatric Neuroblastoma

**DOI:** 10.1515/med-2019-0109

**Published:** 2019-12-31

**Authors:** Robin Wachowiak, Steffi Mayer, Anne Suttkus, Illya Martynov, Martin Lacher, Nathaniel Melling, Jakob R. Izbicki, Michael Tachezy

**Affiliations:** 1 Department of Pediatric Surgery, University Hospital Leipzig, Liebigstrasse 20 A, 04103 Leipzig, Germany; 2 Department of General, Visceral and Thoracic Surgery, University Medical Center Hamburg Eppendorf, Hamburg, 20246, Germany

**Keywords:** CHL1, NrCAM, Neuroblastoma, Immunohistochemistry, Tumor markers, Neuropathology

## Abstract

**Background:**

Neural cell adhesion molecules like close homolog of L1 protein (CHL1) and neuronal glia related cell adhesion molecule (NrCAM) play an important role in development and regeneration of the central nervous system. However, they are also associated with cancerogenesis and progression in adult malignancies, thus gain increasing importance in cancer research. We therefore studied the expression of CHL1 and NrCAM according to the course of disease in children with neuroblastoma.

**Methods:**

CHL1 and NrCAM expression levels were histologically assessed by tissue microarrays from surgically resected neuroblastoma specimens of 56 children. Expression of both markers was correlated to demographics as well as clinical data including metastatic dissemination and survival.

**Results:**

CHL1 was expressed in 9% and NrCAM in 51% of neuroblastoma tissue samples. Expression of CHL1 was higher in patients with low Hughes grade 1a/b (p=0.01). NrCAM was more often detected in patients with a low International Staging System (INSS) score 1/2 (p=0.04).

**Conclusion:**

CHL1 and NrCAM expression was associated with low-grade pediatric neuroblastoma. These adhesion molecules may play a role in early tumor development of neuroblastoma.

## 1 Introduction

Neuroblastoma is an embryonic malignancy deriving from neural crest cells that undergo rapid differentiation during fetal development. As the transition from normal to malignant tissue can occur in multiple steps, its phenotype is highly heterogeneous [1]. Although progress has been made in the treatment of neuroblastoma, the outcome of children at high risk remains poor with a long-term survival as low as 50 % [2]. Different parameters such as age, stage and chromosomal aberrations have an impact on prognosis. Still, there is an ongoing need for tumor markers, which allow a better determination of the individual prognosis and therapeutic monitoring especially in high risk patients.

Recently, cell adhesion molecules (CAMs) have been implicated in the processes of malignant transformation and progression [3]. In particular, members of the immunoglobulin superfamily L1, including transmembrane proteins L1CAM, close homologue of L1 (CHL1), neuron glia related CAM (NrCAM) and neurofascin have been identified as important factors [4, 5]. Various studies emphasized the importance of L1CAMs in neuronal migration and survival, axon outgrowth, synaptic plasticity, and regeneration, resulting in a regular development and homoeostasis of the central nervous system (CNS) [6]. Alterations in the expression and function of CAMs, especially L1, CHL1 and NrCAM, have been associated with pathological processes leading to different malignancies including melanoma, prostate and colon cancer [7, 8, 9, 10, 11, 12]. CHL1 can be down- or upregulated in human cancer genesis [13]. An increased expression of NrCAM has been correlated to metastatic development and tumorigenesis in different human cancers [7, 14, 15]. Moreover, overexpression of NrCAM elevates cancer cell motility and invasiveness *in vitro* and has been linked to a poor prognosis in adult patients [7, 14]. Likewise, overexpression of L1 in adults correlates to tumor progression and metastatic dissemination in glioma, melanoma, ovarial and colon carcinomas [12]. In contrast, an increased expression of NrCAM and L1 in gene array analyses has been associated with a favorable outcome in pediatric neuroblastoma [16, 17].

Taken together, members of the immunoglobulin superfamily L1, which share a similar structure with a 35-45% homology, might serve as interesting prognostic markers in neuroblastoma. The aim of the study was to investigate members of the L1 family with regards to their diagnostic and prognostic potential in this pediatric tumor. We therefore determined the expression of CHL1 and NrCAM by immunohistochemistry in a neuroblastoma tissue microarray and correlated it to the individual course of disease.

## 2 Material and Methods

### 2.1 Study design

The study was approved by the Ethics Committee of the Chamber of Physicians in Hamburg, Germany. The research related to human has been complied with all relevant national regulations, institutional policies and in accordance to the tenets of the Helsinki Declaration. Written informed consent was obtained from all parents for investigation of resected neuroblastoma tissue samples. Pediatric patients who underwent surgical treatment of neuroblastoma at the University Medical Center Hamburg Eppendorf between November 1999 and October 2004 were included. No preselection was performed and none of the children was pretreated. Clinical and pathological data included the International Neuroblastoma Staging System (INSS), histological grade (according to Hughes), N-myc amplification, loss of heterozygosity of chromosome 1p (LOH 1p), age at diagnosis, sex, metastatic dissemination and event free as well as overall survival.

### 2.2 Tissue Microarray

Pediatric neuroblastoma tissues were fixed in 4% buffered formalin and embedded in paraffin as described previously [18]. Hematoxylin-eosin stained sections were cut from primary tumor blocks, containing representative tumor regions. Afterwards, tissue cylinders with a diameter of 600 μm were used to stamp out selected sections of the original donor block. These were arrayed on a new paraffin block using a semi-automated tissue arrayer. Subsequently, 5 μm slides of the complete tissue microarray (TMA) were cut using the paraffin sectioning aid system (Instrumentics, Hackensack, NJ, USA).

### 2.3 Immunohistochemistry

For immunohistochemistry, 5-μm sections were placed on precoated slides (3-triethoxysilylpropylamin; Merck, Darmstadt, Germany), deparaffinized and exposed to heat-induced antigen retrieval for 5 minutes in an autoclave at 121°C in Tris-EDTA-Citrate buffer, pH 7.8. Afterwards, the primary antibody either specific for CHL1 (goat, polyclonal antibody: AF2126, R&D Systems, MN, USA) or NrCAM (goat anti-human NrCAM antibody: AF2034, R&D Systems, MN, USA,) was applied at 37°C and pH 9.1 for 60 minutes. Bound antibodies were then visualized using the EnVision Kit (Dako, Glostrup, Denmark) according to the manufacturer’s directions. Unaffected pancreatic tissue served as positive and lymphoid as negative controls.

### 2.4 Quantification of staining intensities

Staining intensities of positive tumor cells were assessed as described recently [19]. In brief, lack of staining was defined negative, while weak, moderate and strong staining were defined positive. Labeled sections were analyzed by two independent investigators (RW and MT) that were blinded to the patient’s identity or clinical status. A pathologist was involved in cases of discrepancy to reach a consensus.

### 2.5 Statistical Analysis

Statistical analysis was performed by SPSS for Windows version 11.5 (SPSS, IBM Corporation, NY, USA) and Graph-Pad Prism (Version 7.04, GraphPad Software, Inc., San Diego, CA, USA). Chi square test was used to compare categorical variables, Fischer’s exact test to compare odds between two groups and Kruskal-Wallis test for comparisons of continuous variables. Categorical variables are expressed as frequency and percentage; continuous variables are represented as medians with maximum and minimum or as means with standard deviation. Kaplan-Meier survival curves were analyzed using the log-rank test. Significance level was set as p<0.05.

## 3 Results

### 3.1 Study population

A total of 56 children (24 female and 32 male) who underwent surgical resection of neuroblastoma were included in the study. Median age at the time of diagnosis was 30 months. The tumor was localized adrenally in 34 (61%) and non-adrenally in 22 (39%) patients. 28 patients (50%) had metastatic dissemination at the time of operation. Median follow-up of all patients was 72 months. During the observation period, six children (11%) died due to the underlying disease.

### 3.2 Expression of CHL1

**Figure 1:**
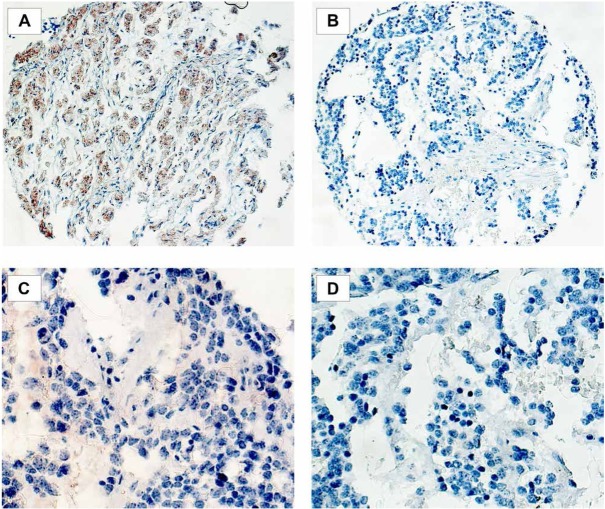
Expression of CHL1 and NrCAM in pediatric neuroblastoma. Representative examples of CHL1 positive (A) and CHL-1 negative (B) (magnification x100) as well as NrCAM positive (C) and NrCAM negative (D) immunostaining (magnification x200).

**Figure 2:**
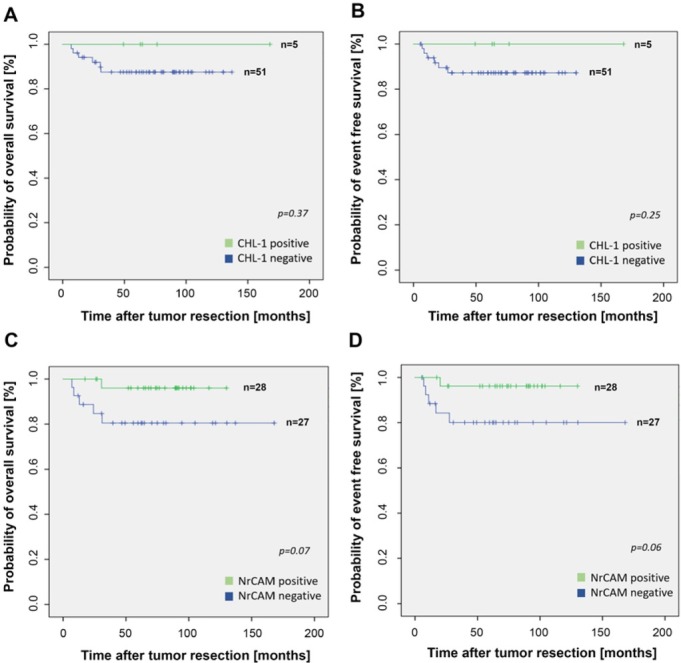
Kaplan-Meier survival curves for overall and event-free survival. No association was found for CHL1-expression (A/B). Survival rates were better by trend in children with NrCAM positive tumors (C/D) but without statistical significance (p=0.07 and p=0.06).

Immunohistochemistry revealed a heterogonous staining pattern of CHL1 inside cancerous lesions with predominant membranous expression. Expression of CHL1 was detected in five (9%) samples (Fig. 1A /1B). Each CHL1 positive tumor was classified Hughes grade 1, which differed significantly from CHL1 negative samples (p=0.01). In CHL1 negative samples, Hughes grade 1 (n=17; 33%), 2 (n=15; 29%) and 3 (n=19; 37%) were equally distributed (p>0.05). Comparing CHL1 positive and negative tumor samples, no significant differences regarding INSS stages, n-MYC amplification as well as 1p mutation status were found. Overall (84.2 vs. 69.0 months) and event free (84.3 months vs. 64.0 months) survival of the children did not differ for CHL1 positive and negative samples (Fig. 2 A/B,

Tab. 1). Subgroup analysis of overall survival for children with metastatic or non-metastatic tumors as well as adrenally or non-adrenally tumors revealed no statistical difference *(data not shown)*.

**Table 1:** CHL1 expression as well as clinical, pathologic and molecular characteristics of the analysed neuroblastoma tissue samples. Statistical analyses by using cross-tables, two-sided Fisher´s and Chi-squared test.

Variable		Number of patients	CHL1 expression		p-value
			positive	negative	
Total		56	5 (9%)	51 (91%)	
age	≤ 1 year	17	1 (6%)	16 (94%)	ns
	> 1 year	39	4 (10%)	35 (90%)	
sex	female	24 (43%)	3 (12%)	21 (88%)	ns
	male	32 (57%)	2 (6%)	30 (94%)	
	1	23 (41%)	2 (9%)	21 (91%)	
	2	13 (23%)	1 (8%)	12 (92%)	
INSS	3	7 (13%)	1 (14%)	6 (86%)	ns
	4	9 (16%)	1 (11%)	8 (89%)	
	4s	4 (7%)	0 (0%)	4 (100%)	
	1a/b	22 (39%)	5 (23%)	17 (77%)	
Hughes grade	2	15 (27%)	0 (0%)	15 (100%)	0.01
	3	19 (34%)	0 (0%)	19 (100%)	
n-MYC amplification	positive	7 (12%)	0 (0%)	7 (100%)	
	negative	49 (88%)	5 (10%)	44 (90%)	ns
LOH1p detection	positive	9 (21%))	1 (11%)	8 (89%)	ns
	negative	35 (79%)	2 (6%)	33 (94%)	

INSS: International Neuroblastoma Staging System

### 3.3 Expression of NrCAM

Likewise, a heterogeneous distribution with prevalent membranous staining was observed after immunostaining for NrCAM. 28 (51%) of the samples showed a positive NrCAM expression (Fig. 1C/1D). Patients with NrCAM positive and negative tissue differed significantly in INSS stages (p=0.04). NrCAM positive tumors were pre-dominantly detected in children with earlier INSS stages (stages 1 and 2) as compared to NrCAM negative samples. No significant differences in Hughes grades, n-MYC amplification and 1p mutation could be found (Tab. 2). Overall (76.0 months vs. 65.3 months) and event free (72.6 months vs. 60.0 months) survival rates were better by trend in children with NrCAM positive tumors but without statistical significance (p=0.07 and p=0.06; Fig. 2 C/D). The subgroup analysis revealed a significantly better overall survival for NrCAM positive tumors (73.8 months) compared to NrCAM negative tumors (65.7 months) in children with metastases (p=0.04). Likewise, patients with adrenal tumors had a significantly longer overall survival with NrCAM positive tissue (81.8 months vs. 60.9 months, p= 0.04). The overall survival comparing NrCAM positive and negative tissue of non-adrenal as well as non-metastatic disease revealed no significant differences *(data not shown)*.

**Table 2:** NrCAM expression as well as clinical, pathologic and molecular characteristics of the analysed neuroblastoma tissue samples. Statistical analyses by using cross-tables, two-sided Fisher´s and Chi-squared test.

Variable		Number of patients	NrCAM expression		p-value
			positive	negative	
Total		55	28 (51%)	27 (49%)	
age	≤ 1 year	17	9 (53%)	8 (48%)	ns
	> 1 year	38	19 (50%)	19 (50%)	
sex	female	23 (42%)	12 (52%)	11 (48%)	ns
	male	32 (58%)	16 (50%)	16 (50%)	
	1	23 (42%)	11 (48%)	12 (52%)	
	2	12 (22%)	9 (75%)	3 (25%)	
INSS	3	7 (13%)	4 (57%)	3 (43%)	0.04
	4	9 (16%)	1 (11%)	8 (89%)	
	4s	4 (7%)	3 (75%)	1 (25%)	
	1a/b	21 (38%)	10 (48%)	11 (52%)	
Hughes grade	2	15 (27%)	11 (73%)	4 (27%)	ns
	3	19 (35%)	7 (37%)	12 (63%)	
n-MYC amplification	positive	7 (13%)	2 (29%)	5 (71%)	ns
	negative	48 (87%)	26 (54%)	22 (46%)	
LOH1p detection	positive	9 (21%)	4 (44%)	5 (56%)	ns
	negative	34 (79%)	20 (59%)	14 (41%)	

INSS: International Neuroblastoma Staging System

## 4 Discussion

In the present study, the protein expression of CHL1 and NrCAM in pediatric neuroblastoma in correlation to clinical and survival data were analyzed. CHL1 was spotted in 9% of the patients, each classified as Hughes grade 1a/b. NrCAM was detected in 51% of the cases predominantly in earlier INSS stages 1/2.

Expression of CHL1 as well as NrCAM has already been studied in neuroblastoma before. Lastowska et al. found an overall downregulation of CHL1 in 28 tumor samples that did not impact on the individual prognosis [20]. Conversely, low CHL1 gene expression in 417 neuroblastoma samples was significantly correlated to a reduced overall survival [21]. That parallels our findings of positive protein expression of CHL1 in low tumor grades Hughes 1a/b by immunohistochemistry only. Thus, expression of CHL1 may be associated with high risk, immature neuroblastoma.

Accordingly, an increased NrCAM gene detection has been reported for low risk neuroblastomas with favorable outcome before [17]. In our study, NrCAM protein expression was detected in 51% of patients, predominantly in children with low risk INSS stage 1 and 2. We also found a trend to a better event free and overall survival for children with NrCAM positive tumors [17]. Thus, the tumor may initially still be able to translate and transcribe NrCAM, but loses this ability within progression. Moreover, the survival analysis using subgroups revealed a significantly better overall survival for patients with NrCAM positive tumors in the subgroup of metastatic tumors as well as for patients with adrenal tumors. These results strengthen the findings that children with NrCAM positive tumors have a favorable outcome even if metastases are detected.

Adhesion molecules of the L1 superfamily are typically found in normal neuronal tissue. NrCAM is expressed in the developing as well as adult organism and can be detected in the central as well as peripheral nervous system [22]. It regulates axonal growth and guidance during development. In the mature brain, NrCAM can be detected at the nodes of Ranvier, suggesting a relevant role in synaptic signaling [3, 22]. CHL1 is found in neuronal development regulating axon growth as well as in cell migration in children and to a lesser extent in adult tissue, predominantly during regeneration processes [23]. The downregulation and -expression of CAMs in high grade neuroblastoma tissue may depict the loss of normal function of the originating neuronal tissue. Thus, the expression of CHL1, NrCAM, L1CAM, and ALCAM may be associated with a less aggressive tumor and, as a consequence, with a better prognosis [16, 24, 25]. However, mechanisms of induction, expression, and intracellular signaling of adhesions molecules especially during tumorigenesis have not been understood yet.

The gene expression of NrCAM and CHL1 has also been studied in adult tumors of various entities with a divergent prognosis. CHL1 was down-regulated in colon, stomach, bladder, or pancreatic cancer but upregulated in lung, liver prostate, and cervix cancers [13, 26, 27]. In contrast, higher levels of NrCAM in tumorous tissue deriving from glioblastoma, pancreatic, and colon cancer as well as papillary thyroid carcinoma were associated with a poorer prognosis [14, 15, 28, 29]. These conflictive results for children and adults are in line with previous findings of our group. We found a more favorable outcome for upregulated cell adhesion molecules L1 and ALCAM in pediatric neuroblastoma patients, while higher expression in adult cancer patients was correlated to poor survival [16, 24, 30]. This might be due to the different tumor entities in pediatric neuroblastoma versus malignancies in adulthood. Interestingly, no significant association between survival and the expression of L1 and NrCAM in other embryonic tumors like rhabdomyosarcoma and Wilms tumor could be found by others [31, 32]. In contrast, in CNS tumors like astrocytoma as well as ependymoma, overexpression of L1 or NrCAM was correlated with a poor prognosis [33, 34, 35, 36]. Thus, the underlying embryonic or non-embryonic ethiopathogenesis may impede the role of CAMs in tumorigenesis. However, their value as prognostic markers has not been fully understood yet [13].

In conclusion, CHL1 and NrCAM as members of the L1 superfamily may play an important role in early tumor development. However, larger prospective studies are warranted to better understand the role of adhesion molecules in neuroblastoma and other embryonic tumors in children and adolescents.

## References

[1] Shohet J, Foster J (2017). Neuroblastoma. Bmj.

[2] Pinto NR, Applebaum MA, Volchenboum SL, Matthay KK, London WB, Ambros PF (2015). Advances in Risk Classification and Treatment Strategies for Neuroblastoma. J Clin Oncol.

[3] Schmid RS, Maness PF (2008). L1 and NCAM adhesion molecules as signaling coreceptors in neuronal migration and process outgrowth. Curr Opin Neurobiol.

[4] Herron LR, Hill M, Davey F, Gunn-Moore FJ (2009). The intracellular interactions of the L1 family of cell adhesion molecules. Biochem J.

[5] Hortsch M (1996). The L1 family of neural cell adhesion molecules: old proteins performing new tricks. Neuron.

[6] Maness PF, Schachner M (2007). Neural recognition molecules of the immunoglobulin superfamily: signaling transducers of axon guidance and neuronal migration. Nat Neurosci.

[7] Liu X, Mazanek P, Dam V, Wang Q, Zhao H, Guo R (2008). Deregulated Wnt/beta-catenin program in high-risk neuroblastomas without MYCN amplification. Oncogene.

[8] Manderson EN, Birch AH, Shen Z, Mes-Masson AM, Provencher D, Tonin PN (2009). Molecular genetic analysis of a cell adhesion molecule with homology to L1CAM, contactin 6, and contactin 4 candidate chromosome 3p26pter tumor suppressor genes in ovarian cancer. Int J Gynecol Cancer.

[9] Rokman A, Baffoe-Bonnie AB, Gillanders E, Fredriksson H, Autio V, Ikonen T (2005). Hereditary prostate cancer in Finland: fine-mapping validates 3p26 as a major predisposition locus. Hum Genet.

[10] Ross DT, Scherf U, Eisen MB, Perou CM, Rees C, Spellman P (2000). Systematic variation in gene expression patterns in human cancer cell lines. Nat Genet.

[11] Wei MH, Karavanova I, Ivanov SV, Popescu NC, Keck CL, Pack S (1998). In silico-initiated cloning and molecular characterization of a novel human member of the L1 gene family of neural cell adhesion molecules. Hum Genet.

[12] Hua T, Liu S, Xin X, Jin Z, Liu Q, Chi S (2016). Prognostic significance of L1 cell adhesion molecule in cancer patients: A systematic review and meta-analysis. Oncotarget.

[13] Senchenko VN, Krasnov GS, Dmitriev AA, Kudryavtseva AV, Anedchenko EA, Braga EA (2011). Differential expression of CHL1 gene during development of major human cancers. PLoS One.

[14] Chan JY, Ong CW, Salto-Tellez M (2011). Overexpression of neurone glial-related cell adhesion molecule is an independent predictor of poor prognosis in advanced colorectal cancer. Cancer Sci.

[15] Sehgal A, Boynton AL, Young RF, Vermeulen SS, Yonemura KS, Kohler EP (1998). Cell adhesion molecule Nr-CAM is over-expressed in human brain tumors. Int J Cancer.

[16] Wachowiak R, Fiegel HC, Kaifi JT, Quaas A, Krickhahn A, Schurr PG (2007). L1 is associated with favorable outcome in neuroblastomas in contrast to adult tumors. Ann Surg Oncol.

[17] Zage PE, Louis CU, Cohn SL (2012). New aspects of neuroblastoma treatment: ASPHO 2011 symposium review. Pediatric blood & cancer.

[18] Reichelt U, Duesedau P, Tsourlakis M, Quaas A, Link BC, Schurr PG (2007). Frequent homogeneous HER-2 amplification in primary and metastatic adenocarcinoma of the esophagus. Modern pathology : an official journal of the United States and Canadian Academy of Pathology, Inc.

[19] Tachezy M, Zander H, Marx AH, Stahl PR, Gebauer F, Izbicki JR (2012). ALCAM (CD166) expression and serum levels in pancreatic cancer. PloS one.

[20] Lastowska M, Viprey V, Santibanez-Koref M, Wappler I, Peters H, Cullinane C (2007). Identification of candidate genes involved in neuroblastoma progression by combining genomic and expression microarrays with survival data. Oncogene.

[21] Pezzolo A, Sementa AR, Lerone M, Morini M, Ognibene M, Defferrari R (2017). Constitutional 3p26.3 terminal microdeletion in an adolescent with neuroblastoma. Cancer Biol Ther.

[22] Sakurai T (2012). The role of NrCAM in neural development and disorders--beyond a simple glue in the brain. Mol Cell Neurosci.

[23] Irintchev A, Schachner M (2012). The injured and regenerating nervous system: immunoglobulin superfamily members as key players. The Neuroscientist : a review journal bringing neurobiology, neurology and psychiatry.

[24] Wachowiak R, Rawnaq T, Metzger R, Quaas A, Fiegel H, Kahler N (2008). Universal expression of cell adhesion molecule NCAM in neuroblastoma in contrast to L1: implications for different roles in tumor biology of neuroblastoma?. Pediatr Surg Int.

[25] Hillenbrand R, Molthagen M, Montag D, Schachner M (1999). The close homologue of the neural adhesion molecule L1 (CHL1): patterns of expression and promotion of neurite outgrowth by heterophilic interactions. The European journal of neuroscience.

[26] He LH, Ma Q, Shi YH, Ge J, Zhao HM, Li SF (2013). CHL1 is involved in human breast tumorigenesis and progression. Biochem Biophys Res Commun.

[27] Zhu H, Fang J, Zhang J, Zhao Z, Liu L, Wang J (2014). miR-182 targets CHL1 and controls tumor growth and invasion in papillary thyroid carcinoma. Biochem Biophys Res Commun.

[28] Dhodapkar KM, Friedlander D, Scholes J, Grumet M (2001). Differential expression of the cell-adhesion molecule Nr-CAM in hyperplastic and neoplastic human pancreatic tissue. Hum Pathol.

[29] Gorka B, Skubis-Zegadlo J, Mikula M, Bardadin K, Paliczka E, Czarnocka B (2007). NrCAM, a neuronal system cell-adhesion molecule, is induced in papillary thyroid carcinomas. Br J Cancer.

[30] Wachowiak R, Mayer S, Kaifi J, Gebauer F, Izbicki JR, Lacher M (2016). Prognostic Impact of Activated Leucocyte Cell Adhesion Molecule (ALCAM/CD166) in Infantile Neuroblastoma. Anticancer research.

[31] Corbin M, de Reynies A, Rickman DS, Berrebi D, Boccon-Gibod L, Cohen-Gogo S (2009). WNT/beta-catenin pathway activation in Wilms tumors: a unifying mechanism with multiple entries?. Genes, chromosomes & cancer.

[32] Inaguma S, Wang Z, Lasota JP, Miettinen MM (2016). Expression of neural cell adhesion molecule L1 (CD171) in neuroectodermal and other tumors: An immunohistochemical study of 5155 tumors and critical evaluation of CD171 prognostic value in gastrointestinal stromal tumors. Oncotarget.

[33] Araki A VJ, Gojo J, Chocholous M, Kiss A, Lotz G SZ, Garami M, Antonelli M, Slavc I, Czech T HB, Haberler C (2015). Molecular Markers and their prognostic Impact in Pediatric Ependymomas. Neuro Oncology.

[34] Kernagis D DM, McLendon R, Grant GA (2012). L1CAM as a Marker of an Aggressive Tumor Phenotype in Children with Juvenile Pilocytic Astrocytoma. Neurosurgery.

[35] Lukashova-v Zangen I, Kneitz S, Monoranu CM, Rutkowski S, Hinkes B, Vince GH (2007). Ependymoma gene expression profiles associated with histological subtype, proliferation, and patient survival. Acta Neuropathol.

[36] Williams RD, Hing SN, Greer BT, Whiteford CC, Wei JS, Natrajan R (2004). Prognostic classification of relapsing favorable histology Wilms tumor using cDNA microarray expression profiling and support vector machines. Genes, chromosomes & cancer.

